# Editorial: Pharmaceutical care and wellness of diabetes

**DOI:** 10.3389/fcdhc.2026.1910046

**Published:** 2026-06-30

**Authors:** Havagiray R. Chitme, Mirza Muhammad Faran Ashraf Baig, Ajay Kumar Gupta

**Affiliations:** 1 Amity Institute of Pharmacy, Amity University, Noida, Uttar Pradesh, India; 2 Faculty of Medicine, The Chinese University of Hong Kong (CUHK), Hong Kong, Hong Kong SAR, China; 3 School of Pharmaceutical Sciences, Chhatrapati Shahu Ji Maharaj University, Kanpur, India

**Keywords:** Ayurveda, diabetes, diabetic complications, Indian system of medicine, Thai medicine, traditional Chinese medicine

The management and treatment of diabetes mellitus (DM) and its complications remain a global health challenge, demanding the development of innovative and integrative team-based approaches. Ethnomedicine, rooted in centuries-old traditional medicine systems, offers a wealth of knowledge about bioactive compounds and therapeutic strategies that could offer safe, effective, and culturally adaptable options for managing diabetes and its associated complications. In recent years, ethnomedicine and other traditional medicine systems have garnered increasing attention for their potential to complement modern medical interventions both through effectively managing and treating DM and in improving overall wellbeing.

This Research Topic, titled “Pharmaceutical Care and Wellness of Diabetes”, aims to bridge the gap between traditional medicine systems and contemporary science by collating research, reviews, and meta-analyses that scientifically validate the potential role of ethnomedicines in bringing wellness to diabetic care. The collection of studies in this issue emphasize the integration of ancient sciences with current medical practices to develop novel therapeutic approaches using safe and efficacious novel mechanisms of action.

We have included a broad spectrum of studies exploring the mechanisms of action, efficacy, safety, and clinical outcomes of alternative, complementary, herbal, Ayurvedic, Chinese, native, and ethnic therapies related to diabetes management. Furthermore, the studies cover modern innovative medical methodologies such as *in silico* modelling, computer-aided drug design, and network pharmacology as pivotal tools to deepen our understanding of ethnomedicines. The following papers exemplify this integrative approach, addressing diverse aspects of diabetes care through traditional and modern scientific perspectives.

The systematic review and meta-analysis of Zhang et al. aimed to assess the efficacy and safety of the traditional Chinese medicine Shengjiang powder in combination with conventional therapy for the treatment of diabetic kidney disease. It significantly improved urinary protein, urinary albumin excretion rate, microalbuminuria, blood urea nitrogen, serum creatinine, fasting and 2hPG blood glucose, hemoglobin A1c, total cholesterol, and triglycerides. A Traditional Thai formulation (Sanpinit et al.) was studied for wound-healing properties of a gauze dressing infused with Ya-Samarn-Phlae. The authors demonstrated that the formulation not only accelerated wound closure but also balanced collagen synthesis and reduced oxidative stress, highlighting its potential as an affordable, effective alternative for diabetic wound management—particularly in resource-limited settings. Another Chinese herbal formulation, Huoxue-Jiangtang decoction (Lee et al.), was found to have promising results in treating type 2 DM through lowering fasting blood glucose levels, improving insulin sensitivity, and repairing injured pancreatic islets. It was demonstrated that the molecular mechanism works by activating the PI3K/AKT signaling pathway and increasing GLUT4 protein expression in skeletal muscles to facilitate glucose uptake along with reducing lipid metabolism disorders and antioxidant and anti-inflammatory properties. This research exemplifies how integrating ethnomedicine with modern scientific methodologies can lead to novel therapeutic strategies for chronic diabetic wounds, kidney disease, and inflammation.

The study by Niu et al. clarified the nephroprotective mechanisms of *Trigonella foenum-graecum L.*, which effectively regulates the glycemic index via a modulatory mechanism involving the PI3K-Akt-ERK signaling pathway. It downregulates over-expressed mRNA and protein levels of important intracellular nodes, including MAPK1, MAPK3, AKT1, and PI3K. Beta-boswellic acid (Han et al.) facilitates diabetic wound healing by targeting STAT3 and inhibiting ferroptosis in fibroblasts; the study explored the therapeutic potential of beta-boswellic acid (β-BA) in promoting diabetic wound healing. The authors demonstrated that β-BA exerts its effects by modulating ferroptosis through the regulation of STAT3 signaling pathways. The chemical constituents of *Tripterygium wilfordii* (Gao et al.), celastrol, triptolide, and triptonide, act through TGF-β/Smad, NF-κB, and JAK/STAT to produce anti-inflammatory and anti-tumor activities. Wang et al., presented comprehensive proteomics and metabolomics analyses demonstrating how extracts of *Knoxia roxburghii* significantly downregulated key proteins involved in glucose metabolism, oxidative stress, and pancreatic function, thereby inhibiting gluconeogenesis, reducing oxidative damage, and preserving pancreatic islet architecture. Wang et al. presented a meta-analysis on the efficacy and safety of berberine, a plant-derived isoquinoline alkaloid used in many traditional system including Chinese medicine for treating type 2 DM. They exemplified how modern molecular approaches can uncover the cellular targets of traditional bioactive medicinal compounds extracted from plants as an innovative treatment strategy for diabetes and its associated care.

Zhou et al., explored the gut-skin axis as a pivotal factor in diabetic foot ulcer pathogenesis and healing. By elucidating mechanisms involving short-chain fatty acids, anti-inflammatory pathways, and key signaling cascades, the authors propose an integrated, multi-targeted approach for the management of DFU that further emphasizes the interconnectedness of gut health and skin healing. This work underscores the importance of harnessing traditional medicine within a modern scientific framework to address complex diabetic complications. Type D personality is one of the most difficult psychobehavioral disorders associated with diabetes. One study used the cluster random sampling method, involving 1000 patients with diabetes from multiple tertiary care hospitals. It reported that the prevalence of risk of depression and type D personality in patients with diabetes is increased compared to the general population. It is also mentioned that the social support intervention can help in managing mental health and reducing the risk of depression.

Alenzi et al. described the efficacy of semaglutide in Saudi Arabian patients with type 2 DM over a 12-month period. Mean HbA1c was significantly reduced, along with body weight, and lipid profiles were significantly improved. Multivariate analysis revealed that baseline HbA1c, disease duration, and insulin use were significant predictors of HbA1c change, while insulin use alone was predictive of the magnitude of weight loss. SGLT2 (Cheng et al.) inhibitors were compared with DPP4 inhibitors to understand their relation to diabetic kidney disease. In this retrospective study, SGLT2i treatment prominently reduced eGFR more than DPP4i, irrespective of the prior rate of eGFR. However, no difference was observed in the urinary albumin-to-creatinine ratio between groups. Rea and Ramdass, provided a comprehensive synthesis of preclinical evidence supporting the protective effects of GLP-1 receptor agonists (GLP-1RAs) on pancreatic beta cells; these agonists have attracted a significant interest due to their potential to enhance insulin secretion and preserve beta cell mass. The authors demonstrate that GLP-1RAs consistently reduce beta cell apoptosis, “highlighting their promise as therapeutic agents that may slow disease progression”. These findings reinforce the importance of further longitudinal clinical studies to translate preclinical benefits into effective, long-lasting treatments for DM.

## Conclusion

As we reflect on the diverse and innovative research presented in this Research Topic, it becomes clear that the integration of ethnomedicine with modern scientific approaches holds tremendous promise for advancing diabetes care. From traditional herbal formulations and natural extracts to cutting-edge molecular targeting and computational modelling, these studies exemplify a holistic and multidisciplinary effort to unlock the therapeutic potential of ancient knowledge systems. They not only deepen our understanding of the complex mechanisms underlying diabetic complications but also enable us to develop safer, more effective, and culturally acceptable treatments.

The synergy between traditional wisdom and contemporary science underscores a paradigm shift towards personalized and integrative medicine, “the approach that might be recognizing the value of ethnomedicines while rigorously validating their efficacy and safety”. For instance, the researchers contributing to this Research Topic explored the gut-skin axis, as well as ferroptosis regulation, and the novel use of bioactive compounds. It reveals that the future of diabetes management looks increasingly promising. We hope these articles will inspire further clinical translational studies worldwide to develop approaches that redefine the landscape of diabetes care, promote wellbeing and happiness, and ultimately improve quality of life for the millions of people around the globe affected by this chronic disease.

